# Neurofilament light as a predictive biomarker of unresolved chemotherapy-induced peripheral neuropathy in subjects receiving paclitaxel and carboplatin

**DOI:** 10.1038/s41598-022-18716-5

**Published:** 2022-09-16

**Authors:** B. L. Burgess, E. Cho, L. Honigberg

**Affiliations:** 1 Development Sciences, Genentech Research and Early Development, 1 DNA Way, South San Francisco, CA 94080 USA; 2 Present Address: Alector, South San Francisco, USA; 3 Early Clinical Development, Genentech Research and Early Development, 1 DNA Way, South San Francisco, CA 94080 USA

**Keywords:** Biomarkers, Oncology, Neuroscience

## Abstract

Management of chemotherapy-induced peripheral neuropathy (CIPN) remains a significant challenge in the treatment of cancer. Risk mitigation for CIPN involves preemptive reduction of cumulative dose or reduction of dose intensity upon emergence of symptoms, despite the risk of reduced tumor efficacy. A predictive biomarker for dose-limiting CIPN could improve treatment outcomes by allowing providers to make informed decisions that balance both safety and efficacy. To identify a predictive biomarker of CIPN, markers of neurodegeneration neurofilament-light (NfL), glial fibrillary acidic protein (GFAP), tau and ubiquitin c-terminal hydrolase L1 (UCHL1) were assessed in serum of up to 88 subjects drawn 21 days following the first of 6 treatments with chemotherapeutics paclitaxel and carboplatin. Serum NfL and GFAP were increased with chemotherapy. Further, NfL change predicted subsequent onset of grade 2–3 CIPN during the remainder of the trial (mean treatment duration = 200 days) and trended toward stronger prediction of CIPN that remained unresolved at the end of the study. These results confirm previous reports that serum NfL is increased in CIPN and provide the first evidence that NfL can be used to identify subjects susceptible to dose-limiting paclitaxel and carboplatin induced CIPN prior to onset of symptoms.

## Introduction

Chemotherapy-induced peripheral neuropathy (CIPN) is a common side effect of chemotherapeutic agents used in the treatment of metastatic disease. Peripheral neuropathy (PN) symptoms vary by the agent used, but may include debilitating loss of motor function, cold or heat sensitivities, paresthesia and neuropathic pain leading to reduced quality of life^1,2^. Once emergent, CIPN symptoms can be persistent, taking months or years to resolve following cessation of treatment^1^.

Risk mitigation strategies are employed to reduce the likelihood of severe or long lasting CIPN. Reduction of cumulative dose, either by prospectively limiting the length of treatment or reduction of dose intensity upon emergence of symptoms, may prevent severe or long-lasting disability yet may also compromise efficacy of these important therapies^1,3^. A predictive biomarker of dose-limiting CIPN could be used to personalize chemotherapy dosing regimen, improving both safety and efficacy of these drugs for the treatment of cancer.

To identify biomarkers predictive of CIPN a panel of neurodegeneration markers were measured in subjects receiving chemotherapy as part of a randomized clinical trial^4^. Serum samples were measured for several proteins known to be elevated in various forms of neurological disease or injury: neurofilament-light (NfL), glial fibrillary acidic protein (GFAP), tau and ubiquitin c-terminal hydrolase L1(UCHL1) ^5–7^. NfL in particular has recently been implicated as a biomarker for CIPN^8^. Kim et al. reported that subjects treated with the chemotherapeutic agent oxaliplatin exhibited increased serum NfL consistent with earlier findings in rat models^9,10^. Serum levels of biomarkers were evaluated for an association with CIPN by inferring PN from adverse event (AE) records.

## Results

### Trial population

**Figure 1:**
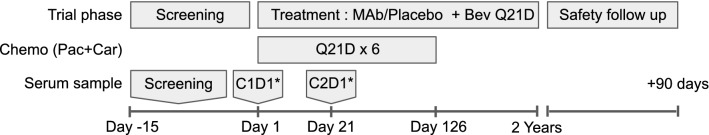
Outline of trial design. Bev: Bevacizumab; Car: Carboplatin; Pac: Paclitaxel, MAb: Experimental antibody; C1D1: Cycle 1 day 1; C2D1: Cycle 2 day 1; Q21D: Dose administered every 21 days; * Sample collected prior to dosing.

Samples were obtained from subjects that received 6 cycles of paclitaxel at 21 day intervals as part of a Ph II clinical trial in non-small cell lung cancer for the investigational therapy Parsatuzumab (Fig. 1)^4^. Up to three serum samples were available for each subject: screening (drawn 1 to 15 days prior to treatment), prior to dosing on cycle 1 day 1 (C1D1) and prior to dosing on day 1 of cycle 2 (C2D1). NfL was measured in all subjects with at least one available baseline and C2D1 sample and that received at least one infusion of chemotherapy prior to C2D1(88 of 103 enrolled; Table 1); GFAP, tau and UCHL1 were measured in a subset of the available samples (55 of 103 enrolled).

**Table 1:** Subject Demographics. Demographics and baseline biomarker levels for all enrolled subjects and subjects included in biomarker analysis.

	Total (N = 103)	Available (N = 88)	Samples unavailable (N = 15)
**Age**			
Mean (SD)	63 (± 9.1)	64 (± 8.9)	58 (± 8.8)
**Sex**			
Female	36 (± 35.0%)	31 (35.2%)	5 (33.3%)
Male	67 (± 65.0%)	57 (± 64.8%)	10 (± 66.7%)
**Race**			
Asian	1 (1.0%)	1 (1.1%)	0 (0%)
Black or African American	2 (1.9%)	2 (2.3%)	0 (0%)
Other race	6 (5%)	6 (6.8%)	0 (0%)
White	94 (91.3%)	79 (89.8%)	15 (100%)
**Treatment**			
Mab + Pac + Car + Bev	52 (50.5%)	43 (48.9%)	9 (60.0%)
Pac + Car + Bev	51 (49.5%)	45 (51.1%)	6 (40.0%)
**Baseline NfL (pg/mL)**			
Mean (SD)	27 (± 30)	27 (± 30)	NA
Missing	15 (14.6%)	0 (0%)	15 (14.6%)
**Baseline GFAP (pg/mL)**			
Mean (SD)	100 (± 56)	100 (± 56)	NA
Missing	48 (46.6%)	33 (37.5)	15 (14.6%)
**Treatment duration (days)**			
Mean (SD)	180 (± 130)	200 (± 130)	75 (± 88)

MAb: Experimental monoclonal antibody; Pac: Paclitaxel, Car: Carboplatin, Bev: Bevacizumab, NA: Not applicable.

### Assessment of CIPN

**Figure 2:**
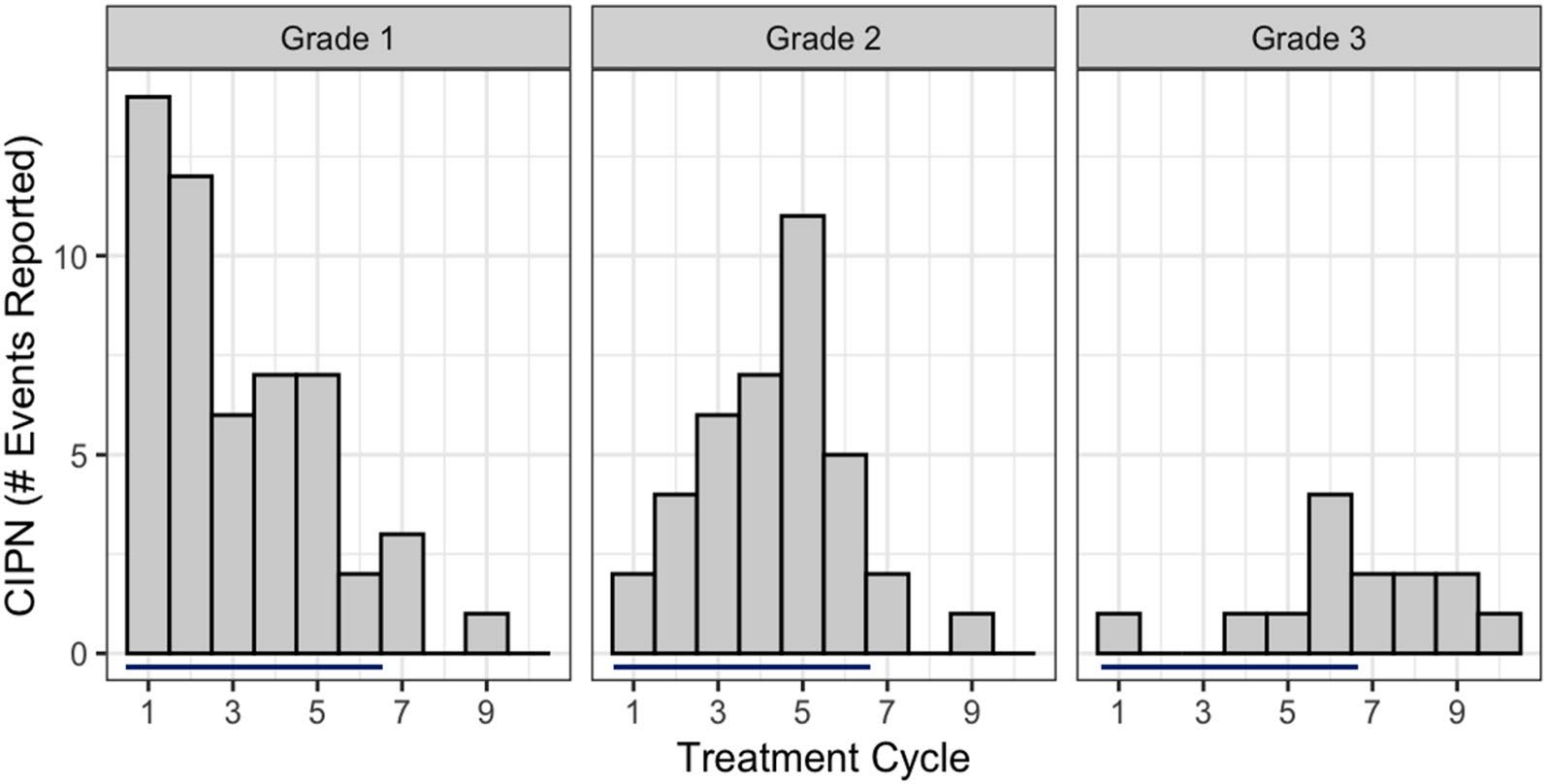
Temporal distribution of CIPN by grade. Bars indicate cycles scheduled for administration of paclitaxel and carboplatin.

CIPN status and grade was assigned using AE records collected as part of the trial. AE preferred terms believed to represent potentially dose-limiting CIPN were selected by an experienced oncologist. As per CTC-NCI guidelines^11^, AEs classified as grade 1 reflect mild symptoms requiring no specific intervention. Grade 2 signified moderate events affecting activities of daily living (ADL). Grade 3 events, the highest grade of CIPN observed in this trial, indicate severe symptoms affecting self-care and ADL. CIPN was reported in 60 subjects (62.5%) in the trial, of which 54 had corresponding NfL data. Lower grade events were more common and tended to occur earlier than higher grades (Fig. 2).

### Effect of chemotherapy on biomarkers of neurodegeneration

**Figure 3:**
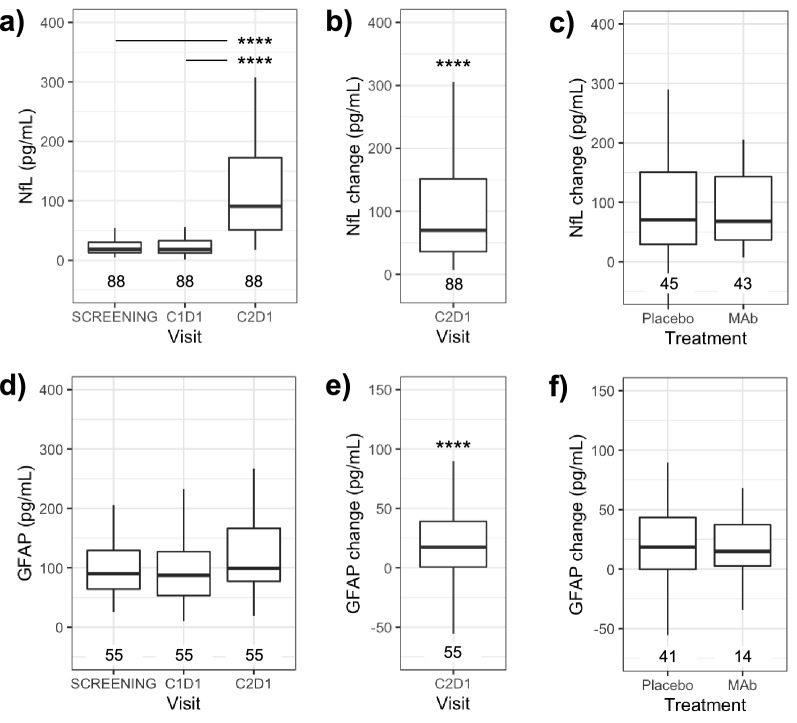
NfL and GFAP in serum are elevated with chemotherapy treatment. (**a**) Serum NfL prior to (screening and C1D1) or 21 days after (C2D1) treatment with paclitaxel and carboplatin. (**b**,**c**) NfL change from baseline, defined as C2D1 levels minus the average of screening and C1D1 levels﻿ (**d**) GFAP levels for all visits or (**e**) GFAP change at C2D1. (**f**) GFAP change by treatment arm.****p < 0.0001 by Dunn’s test: panel (**a**) and (**c**) or Wilcoxon signed rank test: panel (**b**) and (**e**).

In subjects that received chemotherapy, serum NfL at C2D1 (Fig. 3a) was elevated relative to both screening (*p* < 0.0001) and C1D1 (*p* < 0.0001). NfL change at C2D1 was significantly elevated over subject-matched baseline (*p* < 0.0001; Fig. 3b). Treatment with the experimental antibody did not affect NfL change relative to placebo (*p* > 0.05; Fig. 3c), thus treatment and placebo groups were pooled for subsequent analyses.

GFAP change in subjects that received chemotherapy was significantly elevated over subject-matched baseline (*p* < 0.001; Fig. 3d-f). Tau and UCHL1 were both below the lower limit of quantification (LLOQ) in > 50% of samples, consistent with expectations from healthy controls and no trends suggestive of a treatment effect were observed (Dunn’s test *p* > 0.05; data not shown). Results for NfL in the subset of samples with GFAP measures (n = 55) were consistent with the full set with NfL measures (n = 88).

### Neurofilament-light and glial acidic fibrillary protein by CIPN status at C2D1 and C3D1

**Figure 4:**
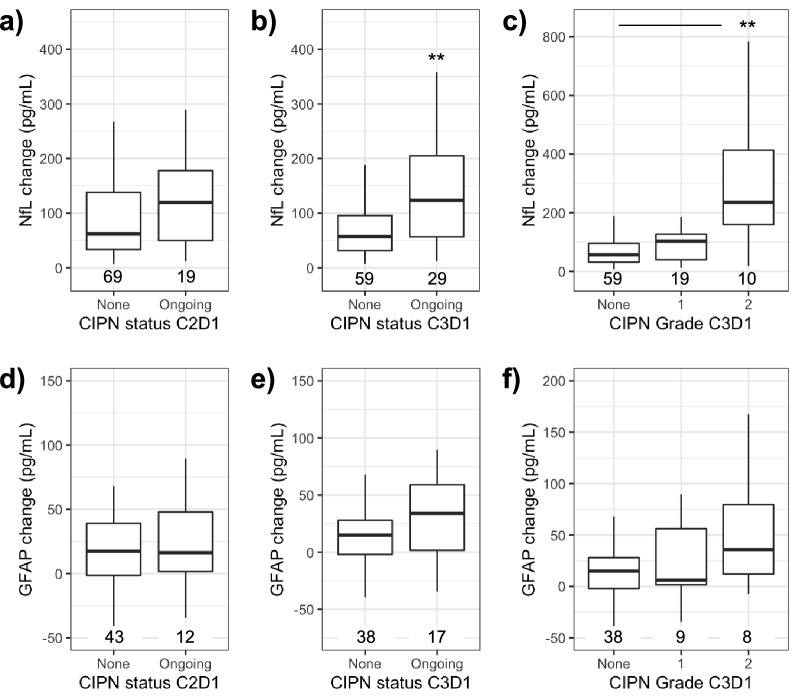
Serum NfL at C2D1 is elevated in subjects reporting CIPN at C3D1 but not C2D1. (**a**) NfL change at C2D1 in subjects with ongoing CIPN at time of sample collection. (**b**) NfL change at C2D1 in subjects with ongoing CIPN at day 1 of cycle 3 (C3D1). (**c**) NfL change at C2D1 by CIPN grade on C3D1. (**d**) GFAP change by CIPN Status at C2D1 or (**e**) C3D1 or (**f**) highest grade of ongoing CIPN at C3D1. **p < 0.01 by Wilcoxon rank sum test panel (**a**,**b**,**d**,**e**) or Dunn’s test panel (**c**) and (**f**).

AE records were used to establish CIPN status and grade as of a given day. At the beginning of cycle 2, C2D1, NfL change in subjects with ongoing CIPN was not different than those without symptoms (*p* > 0.05; Fig. 4a). However, in subjects with ongoing CIPN at the start of the subsequent dosing cycle, day one of cycle 3 (C3D1), NfL change at C2D1 was elevated when compared to subjects without active symptoms at that timepoint (*p* < 0.01; Fig. 4b).

When stratified by highest grade of ongoing CIPN at C3D1, NfL change in subjects with grade 2 CIPN was significantly elevated above those without ongoing CIPN (*p* < 0.01; Fig. 4c). Baseline NfL was not associated with CIPN status at end of cycles 1 or 2 (Dunn’s test *p* > 0.05). In a similar analysis, change in GFAP was not associated with CIPN status or grade at C2D1 or C3D1 (Dunn’s test *p* > 0.05; n = 55; Fig. 4d-f).

The association of NfL and GFAP with CIPN status was repeated using relative change from baseline (fold-change) and results were fully consistent with the results obtained using absolute change (Supplementary Fig. S1). Based on these initial findings, NfL was explored further as a predictive biomarker of CIPN.

### Neurofilament-light as a predictor of future CIPN

**Figure 5:**
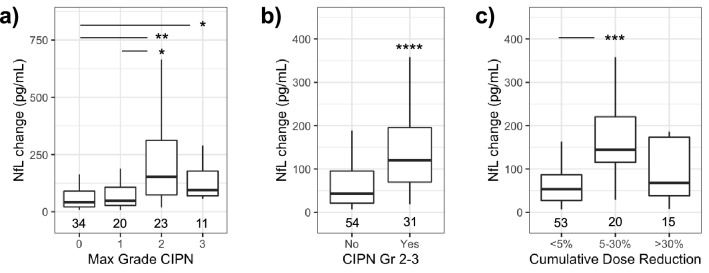
NfL as a predictor of CIPN and paclitaxel dose reduction over duration of the trial. (**a**) Summary of NfL change at C2D1 by highest grade of CIPN reported for all subjects. (**b**) NfL change in subjects that reported grade 2–3 CIPN after C2D1, excluding subjects already reporting such events. (**c**) Reduction of cumulative paclitaxel dose by NfL change *p < 0.05, **p < 0.01, ***p < 0.001, ﻿****p < 0.0001 by Dunn’s test panel (**a**) and (**c**) or Wilcoxon rank sum test panel (**b**).

In this population, frequency and grade of CIPN increased progressively with cumulative dose (Fig. 2) and only a relatively small fraction of the total CIPN reported occurred in cycle 2. Change in NfL at C2D1 was first analyzed by highest grade of CIPN reported over the duration of the study. (Fig. 5a). NfL change was higher in subjects that reported grade 2 CIPN compared to no CIPN (*p* < 0.01) or grade 1 (p < 0.05). Subjects that reported grade 3 CIPN also had higher NfL change at C2D1 relative to subjects that did not report symptoms (*p* < 0.05). Subjects with grade 2 or 3 CIPN had a longer mean treatment time and received more cycles of chemotherapy than other groups (Table 2) yet all subjects had received similar exposure to chemotherapy prior to the NfL timepoint on C2D1. Age was not significantly different among groups (Table 2; one-way ANOVA *p* > 0.05). GFAP levels were not associated with grade of CIPN reported over the duration of the trial (Dunn’s Test *p* > 0.05).

**Table 2:** Subject details summarized by highest grade of CIPN reported.

Max Grade CIPN	Median NfL	Mean age	Time to max grade CIPN	Treatment duration	Cycles of chemo	Cumulative Pac dose reduction	n
	(pg/mL)	(years/SD)	(days)	(days)	(#)	(%)	
0	42	62.4 (9.7)	–	199	4.9	5	34
1	48	61.5 (9.3)	44	183	5.3	10	20
2	153	65.5 (7.6)	64	215	5.5	17	23
3	95	67 (7.1)	117	222	5.6	15	11

Values reflect median NfL change, average age in years (SD), average of time to report of highest grade CIPN, time on study, reduction in cumulative dose of paclitaxel. Chemo: Chemotherapy; Pac: Paclitaxel.

To more precisely test for preditive value, NfL change at C2D1 was also evaluated in an analysis that omitted the three subjects that had already experienced grade 2–3 CIPN at C2D1. In this slightly smaller group, NfL was higher in subjects that reported new grade 2–3 CIPN (median 120 pg/mL; IQR [69.7–196]; n = 31; *p* < 0.0001; Fig. 5b) compared to those that did not (median 42.9 pg/mL; IQR [21.2–95.4]; n = 54). Using receiver operator characteristic (ROC) analysis, NfL change was used to classify subjects that would develop grade 2–3 CIPN with an area under the curve (AUC) of 0.76 95% CI [0.65–0.86)]. Positive and negative predictive values (PPV = 68% and NPV = 66%) were calculated for NfL change at C2D1 to predict future grade 2–3 CIPN (threshold of 73.4 pg/mL). Time between the NfL change timepoint (day 21) and onset of first report of grade 2 or grade 3 CIPN (median 72 days) was not significantly correlated with NfL change (Spearman R = −0.15; p = 0.41).

The association between NfL change and reduction of paclitaxel dose was also examined. Paclitaxel was used as a proxy for both drugs as dose adjustments for paclitaxel were typically similar or larger than carboplatin. Reduction of cumulative dose exhibited a trimodal distribution, with clusters of patients receiving no or minimal (< 5%) reduction, moderate 5–30% reduction, or large reductions of > 30%. Subjects with 5–30% reduction in cumulative dose had a larger NfL change (*p* < 0.001; Fig. 5c) than subjects with < 5% reduction but was not different than subjects with > 30% reduction in cumulative dose (p > 0.05).

To confirm the robustness of the association between change in NfL and the subsequent appearance of CIPN, these analyses were all repeated using relative change from baseline (fold change). Results were consistent with results obtained using absolute change (Supplementary Fig. S2).

### NfL as a predictor of unresolved CIPN

**Figure 6:**
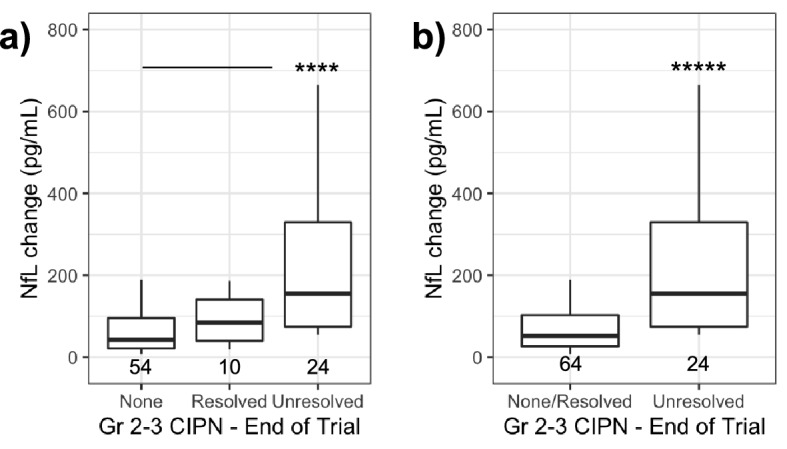
NfL change predicts unresolved grade 2 and 3 CIPN. (**a**) NfL change for CIPN by grade and status at the end of the trial. (**b**) NfL change in unresolved grade 2–3 CIPN compared to all other subjects. ****p < 0.0001; *****p < 0.00001 by Dunn’s test panel (**a**) or Wilcoxon rank sum test panel (**b**).

CIPN that did not resolve over the course of the trial was investigated as an estimate of severe symptoms that may represent dose-limiting toxicity. NfL change at C2D1 was higher in subjects with unresolved grade 2–3 CIPN (Fig. 6a,b) when compared to the group of subjects that did not report grade 2–3 events (p < 0.0001) or the group of subjects that did not report an unresolved grade 2–3 CIPN (p < 0.00001; Fig. 6b). NfL change at C2D1 classified subjects with unresolved grade 2–3 CIPN with an an AUC of 0.81 95% CI [0.72–0.9]. PPV and NPV of ~ 67% were calculated for NfL change at C2D1 to predict unresolved grade 2–3 CIPN at the end of the trial (threshold of 88.0 pg/mL).

A sub-analysis was performed to evaluate the effect of patient drop out prior to the onset of CIPN. This analysis was limited to subjects that remained on treatment until end of cycle 9 (day 189), the final cycle in which an AE associated with CIPN was reported (Fig. 1). Among the subset of individuals enrolled until day 189, NfL change was higher with in those that reported unresolved grade 2–3 CIPN at the end of the trial (median 129 pg/mL; IQR [74.7–550]; n = 10; Dunn’s test *p* < 0.001) compared to the group without unresolved Gr 2–3 CIPN (median 37.1 pg/mL; IQR[19.6–74.4]; n = 32), similar to the main analysis. In this subpopulation NfL change at C2D1 was predictive of unresolved grade 2–3 events with an AUC = 0.86 95% CI [0.74–0.96]) with a PPV of 90% and NPV of 72% using a threshold of 61.7 pg/mL.

### Covariates of NfL and unresolved CIPN

Age is reported to be positively correlated with NfL levels^7,12^ and a risk factor for more severe CIPN in addition to sex and body mass index at baseline (BMI)^13,14^. Logistic regression was used to evaluate age, sex, BMI and baseline NfL in addition to NfL change as co-variates in a relationship with unresolved CIPN. Significant co-linearity (Spearman; *p* < 0.05) was observed between age, NfL change and baseline NfL, and thus these variables were evaluated independently. The logistic regression model with the best fit incorporated parameters NfL change (estimate = 6.48 × 10^–3^, standard error = 2.03 × 10^–3^, z value = 3.20, *p* < 0.01) and BMI at baseline (estimate = 0.139, standard error = 5.72 × 10^–2^, z value = 2.43, *p* < 0.05) as determined by stepwise evaluation of all valid logistic regression models evaluated using AIC. Age, sex or baseline NfL were not statistically significant predictors of unresolved grade 2–3 CIPN in any model (*p* > 0.05).

### NfL change by adverse event term

NfL change was compared across the AE preferred terms used to define CIPN. Specific AE preferred terms were not associated with differences in NfL change (Dunn’s test *p* > 0.05).

### Effect of adverse event term selection on prediction of CIPN

Key analyses were repeated using a standardized list of AE terms associated with PN maintained by the medical dictionary for regulatory activities. All terms in the narrow definition (4 AE preferred terms) were contained in the wider, standardized definition (87 AE preferred terms) of CIPN and produced similar numbers of subjects with at least one report of CIPN (wide: any grade = 65, grade 1 = 51, grade 2 = 37, grade 3 = 14; narrow: any grade = 60, grade 1 = 42, grade 2 = 34, grade 3 = 13). An analysis performed using the standardized wide definition of CIPN produced results that were consistent with those obtained using the narrower criteria.

## Discussion

This study provides the first evidence that serum NfL, a well-established biomarker of axonal injury, predicts onset of paclitaxel and carboplatin induced CIPN. Biomarkers of neurodegeneration, NfL, GFAP, tau and UCHL1 were measured in a cohort of up to 88 subjects at baseline and 21 days after the first of six cycles of paclitaxel and carboplatin chemotherapy, administered as therapy for the treatment of non-small cell lung cancer. NfL was elevated over baseline 21 days after the first dose of chemotherapy. However, NfL was specifically elevated in subjects that reported grade 2 or 3 CIPN during subsequent treatment cycles, but was not elevated in subjects with ongoing CIPN at time of sample collection, of which most (16 of 19 cases) were grade 1. NfL measured at day 21 predicted onset of grade 2 or 3 CIPN that had a median onset of day 72. Further, a trend toward stronger prediction was observed in the subset of grade 2–3 CIPN that had not resolved by the end of the trial. These results suggest that serum NfL early in treatment with paclitaxel and carboplatin can identify individuals susceptible to developing higher grades of slow resolving, potentially dose-limiting, CIPN prior to onset of symptoms.

These results confirm previous reports that NfL is increased in neuropathies, including CIPN. Subjects with acquired peripheral neuropathies exhibit elevated serum NfL compared to age matched controls without neurological disease^15^. Additionally, Kim et al. recently reported that serum NfL increased progressively with oxaliplatin chemotherapy between baseline, 3 and 6 months^8^. NfL was preferentially increased in subjects that reported grade 3 CIPN over lower grades or subjects without CIPN, similar to results presented here. NfL changes reported by Kim et al. appear slower to manifest (approximate median NfL change at 3 months = 10 pg/mL, 6 months = 100 pg/mL) compared to present study (median NfL change at 3 weeks = 69.7 pg/mL). This discrepancy may reflect differences in toxicity or rate of symptom onset for the two regimens, consistent with reports of rapid onset of PN following paclitaxel treatment^16^.

Early discontinuation of patients prior to onset of PN symptoms may result in susceptible subjects being misclassified thus decreasing the apparent predictive power of NfL. Early discontinuation was observed in 25% of patients by the median onset of first grade 2–3 event (day 72) and in 52% of patients by the date at which the final event was reported (day 186). Consistent with this effect a sub-analysis of patients that remained enrolled until the end of cycle 10 observed a trend towards a stronger prediction of unresolved grade 2–3 CIPN than the full analysis that did not account for early discontinuation.

A predictive biomarker of susceptibility to dose-limiting CIPN may have several applications. Chemotherapy regimen tailored to individual patients using NfL may allow larger cumulative doses of chemotherapeutics to be administered safely to resistant subjects, thus achieving higher treatment response while susceptible patients could be spared from debilitating side effects. Alternatively, clinical trials for treatment or prevention of CIPN could use NfL to enrich or balance treatment arms with subjects at risk for CIPN, thus reducing the number of patients required.

Biomarkers, GFAP, tau and UCHL1 were not strong markers of paclitaxel and carboplatin induced CIPN in this study. An association between GFAP and chemotherapy was observed, however levels were not associated with ongoing CIPN and did not predict onset of future symptoms, consistent with recently published findings^8^. Levels of tau and UCHL1 were both largely below the LLOQ of the assay, as expected for healthy subjects, suggesting that CIPN did not elicit changes as large or consistent as those observed in indications such as traumatic brain injury or Alzheimer’s disease^5,6,17^.

## Limitations

A key limitation of this study is the reliance on adverse events as a surrogate for more meaningful patient reported outcomes. CTC-NIC AE grades examined in the present study are reported to correlate well with patient reported outcomes at the group level, however, significant variability individual patient level remains^18,19^. Assessment of NfL in conjunction with long term patient outcomes may lead to improved prediction of relevant sequelae.

Another limitation of this study is the reliance on a single timepoint, collected after only 1 of 6 scheduled treatments with chemotherapy. Longitudinal analysis of NfL or use of a later timepoint may result in greater predictive power or be more representative of long-term outcomes than the single timepoint examined in the present study.

The generalizability of the results presented here is limited by several factors. Prevalence of CIPN and time to symptom onset varies by agent, cumulative dose and dose intensity^1^ suggesting that the magnitude or kinetics of NfL change for various therapeutic regimen may differ from the single regimen examined in the present study. Further, carboplatin and paclitaxel are reported to cause PN by distinct mechanisms and it remains unclear which potential mechanism underlies the NfL changes observed in this study^20^. Additionally, co-administration of bevacizumab has been reported to exacerbate CIPN^21^, suggesting that multiple factors not evaluated here may affect the specific relationship between NfL and CIPN.

## Conclusions

We confirm previous findings that serum NfL is elevated in subjects with CIPN and provide the first evidence that NfL changes early in treatment can predict dose-limiting CIPN prior to onset of symptoms in subjects receiving paclitaxel and carboplatin. Further study is required to confirm and refine these observations across other patient populations, chemotherapeutic agents and treatment regimens to refine the utility of NfL in reducing peripheral neuropathy resulting from cancer therapies.

## Methods

### Patient population and trial design

Serum samples were obtained from a subjects with advanced or recurrent non-small cell lung cancer scheduled to receive 6 cycles of paclitaxel (200 mg/m^2^) and carboplatin (AUC 6 mg*min/mL) administered once every 21 days as part of a randomized clinical trial (ClinicalTrials.gov identifier NCT01366131)^4^. Subjects were randomized to receive an experimental antibody specific to epidermal growth factor like domain-7 or placebo plus bevacizumab (15 mg/kg) every 21 days for 24 months or until death or disease progression. Exclusion criteria included clinically suspected or confirmed central nervous system metastases evaluated by computerized tomography or magnetic resonance imaging within 28 days of randomization, history of stroke or transient ischemic attacks within 6 months prior to day 1. No efficacy was associated with the experimental antibody in this trial or others^4,22^. The clinical trial was conducted according to the principles of the Declaration of Helsinki and all applicable laws and regulations of the country where the research was conducted. Study protocol and procedures were first approved by the institutional review board of Hematology Oncology Associates of the Treasure Coast and all subsequent participating clinical sites (Supplementary Table S1) prior to study initiation. All methods were carried out in accordance with relevant guidelines and regulations. Samples and data were analyzed consistent with written informed consent statements obtained from all subjects.

### Definition of CIPN

Adverse events were classified using the Common Toxicity Criteria of the National Cancer Institute version 4.0 (CTC NCI) and included AE Preferred Term, grade, date of onset, date of resolution if applicable and resolution status at time last report. AE Preferred terms used to establish CIPN were Neuropathy Peripheral, Paraesthesia, Peripheral Sensory Neuropathy and Poly Neuropathy. CIPN status and grade on a given timepoint reflects the highest grade of ongoing AE associated with CIPN. Events were recorded for 90 days following the last study treatment or initiation of another anti-cancer therapy. Unresolved CIPN was defined﻿ as events that were unresolved or resolved with sequelae and events classified as resolved included AEs indicated as resolved or resolving.

In a sub-analysis, AE terms associated with PN were obtained from a ﻿standardized list of terms for maintained by the Medical Dictionary for Regulatory Activities (MedDRA). MedDRA terminology is the international medical terminology developed under the auspices of the International Council for Harmonization of Technical Requirements for Pharmaceuticals for Human Use. MedDRA trademark is registered by IFPMA on behalf of ICH.

### Immunoassays

NfL, GFAP, tau and UCHL1 were measured by multiplex high sensitivity immunoassay (Neuro-4-plex B, Quanterix Inc). A subset of subjects (n = 33 / total n = 88) were assayed for NfL only (NfL Advantage, Quanterix Inc). Matched controls (n = 10) had mean error of ≤ 10% between multiplex and single-plex assays. Functional serum LLOQ for NfL = 2.00 pg/mL, GFAP = 37.5 pg/mL, Tau = 37.5 pg/mL, UCHL1 0.5 pg/mL.

### Calculation of NfL change from baseline

NfL change was calculated as the level on C2D1 subtracted by the average available pre-treatment timepoints, screening and C1D1.

### Calculation of positive and negative predictive values

The hypothetical positive and negative predictive value of NfL for the entire trial was simulated using a bootstrap method to account for subjects missing NfL data (n = 15, 12%). Values for NfL change were sampled with replacement from available data to construct populations matching the observed rate of CIPN among all subjects.

### Calculation of paclitaxel dose reduction

Cumulative dose reduction was calculated as the average percent reduction in dose relative to the initial dose for all visits in which the subject remained on chemotherapy.

### Statistics

Data for NfL and GFAP did not conform to a normal distribution therefore analysis was performed using median values and statistical tests suitable for non-parametric data. Statistical tests were performed as two-sided comparisons and reported p values for Dunn’s test were adjusted for multiplicity using the Holm method.

Boxplots reflect median, first and third quartiles and whiskers span all observed values to a maximum distance of 1.5-fold * IQR from the nearest quartile.

All graphics and tables were prepared using R v 3.5.2 (R Core Team, 2018 In Studio v1.1.452 (R Studio Team, 2016). Statistical analyses were conducted using built in packages except for Dunn’s Test included in the FSA package v 0.8.30 (Ogle, D.H., P. Wheeler, and A. Dinno, 2020) and ROC tests conducted using pROC v 1.16.2 (Robin X, et al.,2011).

## Supplementary Information

Supplementary Information 1.

Supplementary Information 2.

## Data Availability

Datasets used to support the findings of this study can be made available upon reasonable request consistent with the Roche data sharing policy. Patient level data may be requested through https://vivli.org.

## References

[1] Flatters SJL, Dougherty PM, Colvin LA (2017). Clinical and preclinical perspectives on chemotherapy-induced peripheral neuropathy (CIPN): A narrative review. Br. J. Anaesth..

[2] Staff NP, Grisold A, Grisold W, Windebank AJ (2017). Chemotherapy-induced peripheral neuropathy: A current review. Ann. Neurol..

[3] Havrilesky LJ, Reiner M, Morrow PK, Watson H, Crawford J (2015). A review of relative dose intensity and survival in patients with metastatic solid tumors. Crit. Rev. Oncol. Hematol..

[4] von Pawel J (2018). Randomized phase II trial of parsatuzumab (anti-EGFL7) or placebo in combination with carboplatin, paclitaxel, and bevacizumab for first-line nonsquamous non-small cell lung cancer. Oncologist.

[5] Papa L (2016). Time course and diagnostic accuracy of glial and neuronal blood biomarkers GFAP and UCH-L1 in a large cohort of trauma patients with and without mild traumatic brain injury. JAMA Neurol..

[6] Gill J, Merchant-Borna K, Jeromin A, Livingston W, Bazarian J (2017). Acute plasma tau relates to prolonged return to play after concussion. Neurology.

[7] Bridel C (2019). Diagnostic value of cerebrospinal fluid neurofilament light protein in neurology: A systematic review and meta-analysis. JAMA Neurol..

[8] Kim SH (2020). Serum neurofilament light chain levels as a biomarker of neuroaxonal injury and severity of oxaliplatin-induced peripheral neuropathy. Sci. Rep..

[9] Meregalli C (2020). Neurofilament light chain: A specific serum biomarker of axonal damage severity in rat models of Chemotherapy-Induced Peripheral Neurotoxicity. Arch. Toxicol..

[10] Meregalli C (2018). Neurofilament light chain as disease biomarker in a rodent model of chemotherapy induced peripheral neuropathy. Exp. Neurol..

[11] Kaplow R, Iyere K (2017). Grading chemotherapy-induced peripheral neuropathy in adults. Nursing.

[12] Khalil M (2020). Serum neurofilament light levels in normal aging and their association with morphologic brain changes. Nat. Commun..

[13] Ghoreishi Z (2018). Risk factors for paclitaxel-induced peripheral neuropathy in patients with breast cancer 11 Medical and Health Sciences 1112 oncology and carcinogenesis. BMC Cancer.

[14] Mizrahi D (2021). Hemoglobin, body mass index, and age as risk factors for paclitaxel- and oxaliplatin-induced peripheral neuropathy. JAMA Netw. Open.

[15] Mariotto S (2018). Serum and cerebrospinal neurofilament light chain levels in patients with acquired peripheral neuropathies. J. Peripher. Nerv. Syst..

[16] Loprinzi CL (2017). Natural history of paclitaxel-associated acute pain syndrome: prospective cohort study NCCTG N08C1. J. Clin. Oncol..

[17] Mattsson N (2016). Plasma tau in Alzheimer disease. Neurology.

[18] Le-Rademacher J (2017). Patient-reported (EORTC QLQ-CIPN20) versus physician-reported (CTCAE) quantification of oxaliplatin- and paclitaxel/carboplatin-induced peripheral neuropathy in NCCTG/Alliance clinical trials. Support. Care Cancer.

[19] Tan AC, McCrary JM, Park SB, Trinh T, Goldstein D (2019). Chemotherapy-induced peripheral neuropathy-patient-reported outcomes compared with NCI-CTCAE grade. Support. Care Cancer.

[20] Zajączkowska R (2019). Mechanisms of chemotherapy-induced peripheral neuropathy. Int. J. Mol. Sci..

[21] Matsuoka A (2016). Bevacizumab exacerbates paclitaxel-induced neuropathy: A retrospective cohort study. PLoS ONE.

[22] García-Carbonero R (2017). Randomized phase II trial of parsatuzumab (Anti-EGFL7) or placebo in combination with FOLFOX and bevacizumab for first-line metastatic colorectal cancer. Oncologist.

