# Supplementary material for: Neurofilament light as a predictive biomarker of unresolved chemotherapy-induced peripheral neuropathy in subjects receiving paclitaxel and carboplatin
Source: Sci Rep. 2022 Sep 16;12:15593. doi: 10.1038/s41598-022-18716-5 (PMC9481642; doi:10.1038/s41598-022-18716-5)
Supplement: Supplementary file 2 — Supplementary Information 2. [file 41598_2022_18716_MOESM2_ESM.docx]

**Supplementary Information**

| **Investigator** | **Study Center** | **Site Number** | **IRB Approval Date** |
| --- | --- | --- | --- |
| Barlesi, Fabrice | CHU Nord | 237345 | 7/25/11 |
|  | Chemin des Bourrely |  |  |
|  | Service d'Oncologie Multidisciplinaire et Innovations thérapeutiques cedex 20 |  |  |
|  | Marseille France 13915 |  |  |
| Belani, Chandra P. | Penn State Milton S. Hershey Medical Center | 234250 | 9/29/11 |
|  | 500 University Drive |  |  |
|  | Penn State Cancer Institute Milton S. Hershey Medical Center |  |  |
|  | Hershey, PA 17033 |  |  |
| Bennouna-Louridi, Jaafar | Centre René Gauducheau - Centre de Lutte contre le cancer Nantes - Atlantique | 234603 | 10/26/11 |
|  | Boulevard Jacques Monod - Saint Herblain Service Oncologie Médicale |  |  |
|  | Site hospitalier Nord - Cedex 1 |  |  |
|  | Nantes France 44805 |  |  |
| Birhiray, Ruemu E. | Hematology-Oncology of Indiana PC 8301 Harcourt Road, Suite 207 | 234223 | 1/17/12 |
|  | Indianapolis, IN 46260 |  |  |
| Böcskei, Csaba | Szent Borbála Kórház Szanatórium út 1-3. | 234595 | 7/14/11 |
|  | II. Tüdögyógyászat |  |  |
|  | Tatabánya Hungary 2800 |  |  |
| Chmielowska, Ewa C. | Regionalne Centrum Onkologii Im. Prof. Lukaszczyka Izabeli Romanowskiej 2 | 234338 | 1/24/12 |
|  | Oddzial Kliniczny Onkologii |  |  |
|  | Bydgoszcz Poland 85-796 |  |  |
| Crequit, Perrine (Replaced Chouaid, Christos) | Hopital Tenon | 234252 | 10/26/11 |
|  | Service de pneumologie Hôpital tenon |  |  |
|  | 4 rue de la Chine |  |  |
|  | Paris France 75020 |  |  |
| Fayette, Jerôme | Centre Léon Bérard - Centre régional de lutte contre le cancer Rhône-Alpes | 234604 | 5/11/12 |
|  | 28 Rue Laennec |  |  |
|  | Service Oncologie médicale Cedex 08 |  |  |
|  | Lyon France 69008 |  |  |
| Gore, Jr., Ira | Birmingham Hematology and Oncology Associates, LLC 100 Pilot Medical Drive, Suite 175 | 236551 | 8/17/11 |
|  | Birmingham, AL 35235 |  |  |
| Hermann, Robert | Northwest Georgia Oncology Center | 234231 | 6/17/11 |
|  | 340 Kennestone Hospital Blvd, Suite 200 |  |  |
|  | Marietta, GA 30060 |  |  |
| Iannotti, Nicholas O. | Hematology Oncology Associates of the Treasure Coast, | 234173 | 5/17/11 |
|  | PA 1871 Southeast Tiffany Avenue, Suite 100 |  |  |
|  | Port St. Lucie, FL 34952 |  |  |
| Juhász, Erzsébet | Országos Korányi TBC és Pulmonológiai Intézet Pihenö út 1. | 234367 | 7/14/11 |
|  | XIV. Pulmonológiai Osztály |  |  |
|  | Budapest Hungary 1121 |  |  |
| Losonczy, György | Semmelweis Egyetem Diósárok út 1/c. | 234255 | 7/14/11 |
|  | Pulmonológiai Klinika |  |  |
|  | Budapest Hungary 1125 |  |  |
| Ervin, Thomas (was replaced by Lunin, Scott) | Florida Cancer Specialists | 237198 | 7/7/11 |
|  | 4612 North Habana Avenue |  |  |
|  | Tampa, FL 33614 |  |  |
| Lunin, Scott | Florida Cancer Specialists | 234429 | 6/28/11 |
|  | 4331 Veronica South Shoemaker Boulevard, Unit 15 |  |  |
|  | Fort Myers, FL 33916 |  |  |
| Madroszyk, Anne | Institut Paoli Calmettes | 234260 | 10/26/11 |
|  | 232 Boulevard Sainte Marguerite Département d'oncologie médicale Cedex 9 |  |  |
|  | Marseille France 13273 |  |  |
| Mazieres, Julien | Hôpital Larrey Université Paul Sabatier Avenue Jean Poulhes TSA 30030 | 234600 | 10/26/11 |
|  | Toulouse France 31059 |  |  |
| Mekhail, Tarek | Cancer Institute of Florida 2501 North Orange Avenue | 234429 | 6/28/11 |
|  | Oncology Clinical Research, Suite 689 |  |  |
|  | Orlando, FL 32804 |  |  |
| Molinier, Olivier | Centre Hospitalier Le Mans 194 av. Rubillard | 244207 | 4/3/12 |
|  | cedex 9 |  |  |
|  | Le Mans France 72037 |  |  |
| Page, Ray D. | Center for Cancer and Blood Disorders 800 West Magnolia Avenue | 236842 | 7/13/11 |
|  | Fort Worth, TX 76104 |  |  |
| Pápai-Székely, Zsolt | Fejér Megyei Szent György Kórház Seregélyesi út 3. | 234328 | 7/14/11 |
|  | I. Pulmonológiai Osztály |  |  |
|  | Székesfehérvár Hungary 8000 |  |  |
| Pavlakis, Nick | Royal North Shore Hospital Pacific Highway | 234239 | 4/3/12 |
|  | St Leonards NSW |  |  |
|  | Australia 2065 |  |  |
| Pittman, Ken | Central Northern Adelaide Health Services Ethics of Human Research Committee (TQEH & LMH) | 237164 | 12/5/11 |
|  | 28 Woodville Road |  |  |
|  | Haematology and Medical Oncology Department, Ward 8B |  |  |
|  | Woodville, SA |  |  |
|  | Australia 5011 |  |  |
| Reck, Martin | LungenClinic Grosshansdorf GmbH Wöhrendamm 80 | 237744 | 2/13/12 |
|  | Großhansdorf Germany 22927 |  |  |
| Roubec, Jaromir | Fakultni nemocnice Ostrava | 234321 | 10/17/11 |
|  | 17. listopadu 1790, Ostrava |  |  |
|  |  |  |  |
|  | Czech Republic 708 52 |  |  |
| Schuler, Martin | Universitätsklinikum Essen Hufelandstrasse 55 | 238511 | 2/13/12 |
|  | WTZ-Ambulanz Innere Klinik - Tumorforschung |  |  |
|  | Essen Germany 45122 |  |  |
| Spigel, David R. | Tennessee Oncology | 234236 | 7/7/11 |
|  | 250 25th Avenue North, Suite 100 |  |  |
|  | Nashville, TN 37203 |  |  |
| Von Pawel, Joachim | Asklepios Fachkliniken GmbH Robert-Koch-Allee 2 | 237746 | 2/13/12 |
|  | Gauting Germany 82131 |  |  |

Supplementary Table S1. List of institutional review boards that approved clinical procedures.
